# Quantitative diffusion MRI biomarkers predict adverse maternal outcomes in placenta accreta spectrum disorders

**DOI:** 10.3389/fmed.2025.1639652

**Published:** 2025-12-10

**Authors:** Tao Lu, Li Wang, Yufu Huang, Yazheng Chen, Mou Li

**Affiliations:** Department of Radiology, Sichuan Provincial People’s Hospital, University of Electronic Science and Technology of China, Chengdu, China

**Keywords:** placenta accreta spectrum disorders, diffusion-weighted MRI, intravoxel incoherent motion, diffusion kurtosis imaging, postpartum hemorrhage

## Abstract

**Introduction:**

This study aimed to investigate the potential of diffusion-weighted imaging (DWI) parameters in predicting adverse maternal outcomes in high-risk patients with placenta accreta spectrum (PAS) disorders.

**Methods:**

We conducted a prospective analysis of 109 patients with PAS, including 15 who experienced adverse maternal outcomes and 94 who did not. All patients underwent DWI, including intravoxel incoherent motion (IVIM) and diffusion kurtosis imaging (DKI). The measured parameters included the apparent diffusion coefficient (ADC), pure diffusion coefficient (D), pseudo-diffusion coefficient (D*), perfusion fraction (f), mean kurtosis (MK), and mean diffusivity (MD). These parameters were compared between the two groups. Logistic regression and receiver operating characteristic (ROC) curve analyses were performed to evaluate the diagnostic performance of DWI parameters in predicting adverse maternal outcomes.

**Results:**

Patients with adverse maternal outcomes exhibited significantly higher MD, D, and D* values but significantly lower MK values (*p* < 0.05). A multivariate regression analysis identified D and the number of prior cesarean deliveries (CDs) as independent predictors of adverse outcomes. The combination of these two factors yielded the highest predictive performance, with an area under the curve (AUC) of 0.766 (95% confidence interval: 0.654–0.878).

**Discussion:**

DWI parameters demonstrated a significant association with adverse maternal outcomes in high-risk PAS patients. The combination of prior CD history and the D parameter may serve as a valuable predictive tool for identifying patients at an increased risk of complications.

## Introduction

Placenta accreta spectrum (PAS) disorders encompass a continuum of pathological placental invasion into the uterine myometrium, classified as placenta accreta, increta, or percreta according to the depth of invasion. Current epidemiological data indicate an incidence of approximately 1 in 533 deliveries, with projections suggesting a rising trend in prevalence over time (1). Any surgical procedure that compromises uterine wall integrity may disrupt normal decidualization, thereby promoting the pathological infiltration of extravillous trophoblasts and deep villous tissue invasion into the myometrium (2).

The strongest known risk factor for placenta accreta spectrum (PAS) disorders is placenta previa, particularly when coexisting with prior cesarean deliveries. Notably, this risk exhibits a dose-dependent relationship with the number of previous cesarean sections (3–9). A previous study demonstrated a notable increase in the incidence of PAS disorders, increasing from 1.7 to 577 cases per 1,000 pregnancies among women with both placenta previa and a history of cesarean delivery. This represents approximately a 340-fold elevation in risk when these two factors coexist (10). Additional established risk factors for PAS disorders include advanced maternal age (typically >35 years) and a history of uterine surgical procedures, including operative hysteroscopy, suction curettage, surgical termination of pregnancy, and endometrial ablation (10–13).

All subtypes of placenta accreta spectrum (PAS) disorders carry a significant risk of life-threatening postpartum hemorrhage, which may precipitate severe secondary complications, including disseminated intravascular coagulopathy, multisystem organ failure, and maternal mortality (14–19). In severe cases of placenta accreta spectrum (PAS) disorders, the invasive placental tissue may extend beyond the uterus to involve adjacent structures such as the urinary bladder, the parametrium, the uterine ligaments, and the ureters. This aggressive form of PAS was associated with significantly higher risks of massive transfusion requirements, urological injuries necessitating complex bladder reconstruction, and critical care admission due to life-threatening hemorrhage (20–22).

Accurate prenatal prediction of adverse maternal outcomes enables comprehensive multidisciplinary team (MDT) counseling and facilitates personalized treatment planning. This approach optimizes care for high-risk PAS patients, ultimately reducing severe maternal morbidity and mortality through timely, targeted interventions.

Ultrasonography (US) remains the primary imaging modality for diagnosing placenta accreta spectrum (PAS) disorders. While magnetic resonance imaging (MRI) has been increasingly adopted in tertiary care centers as a complementary tool, offering advantages such as a wider field-of-view and superior soft tissue contrast, current evidence suggests comparable diagnostic accuracy between these two modalities for PAS detection (23–25). The principal clinical value of MRI lies in its ability to provide prognostic information beyond mere PAS diagnosis. By offering detailed anatomical characterization of placental invasion patterns and surrounding tissue involvement, MRI enables more accurate prediction of potential surgical challenges and hemorrhage risk. This facilitates comprehensive delivery planning and individualized surgical strategy development, particularly in complex cases requiring multidisciplinary management.

Diffusion-weighted imaging (DWI) represents a non-invasive MRI technique capable of quantifying placental function *in vivo* while providing unique biological insights into tissue cellularity, vascularity, and microstructural characteristics. Advanced DWI modeling approaches include intravoxel incoherent motion (IVIM), which independently quantifies perfusion using perfusion fraction (f) and diffusion components using diffusion coefficient (D) and pseudo-diffusion coefficient (D*), respectively, and diffusion kurtosis imaging (DKI), which characterizes tissue cellularity and structural heterogeneity using mean kurtosis (MK), and mean diffusivity (MD). Liu et al. used IVIM to assess placental perfusion in fetal growth-restricted pregnancies and showed that the perfusion fraction was significantly lower in these patients (26). León et al. also used IVIM in PAS patients and found that the perfusion fraction was significantly higher in the patients (27). Our prior investigations utilized IVIM and DKI to predict postpartum hemorrhage >1,000 mL in patients at high risk for PAS disorders. The results revealed that D, D*, f, and MD were positively correlated with the amount of bleeding during delivery, whereas MK was negatively correlated with it (28). These findings underscore the importance of multiparametric DWI assessment, as different parameters from distinct DWI models collectively provide complementary information essential for comprehensive placental evaluation.

This study had two primary objectives: First, to investigate whether significant differences exist in placental functional parameters between PAS patients with and without adverse maternal outcomes. Second, to evaluate the potential for diffusion-weighted imaging (DWI) parameters as predictive biomarkers for adverse maternal outcomes in high-risk PAS cases.

## Materials and methods

This prospective study received approval from our Institutional Review Board, with written informed consent obtained from all participants. From November 2021 to March 2023, we screened 198 consecutive patients who underwent placental MRI, including DWI sequences, at our tertiary care center. Inclusion criteria comprised the following: (1) clinical suspicion of PAS disorders or inconclusive ultrasound findings, (2) singleton viable pregnancy, and (3) appropriate fetal growth for gestational age. Exclusion criteria included the following: (1) major medical comorbidities (chronic hypertension, pre-existing renal disease, or diabetes mellitus), (2) incomplete surgical or outcome data, (3) suspected placental insufficiency, and (4) non-diagnostic MRI quality.

**Figure 1:**
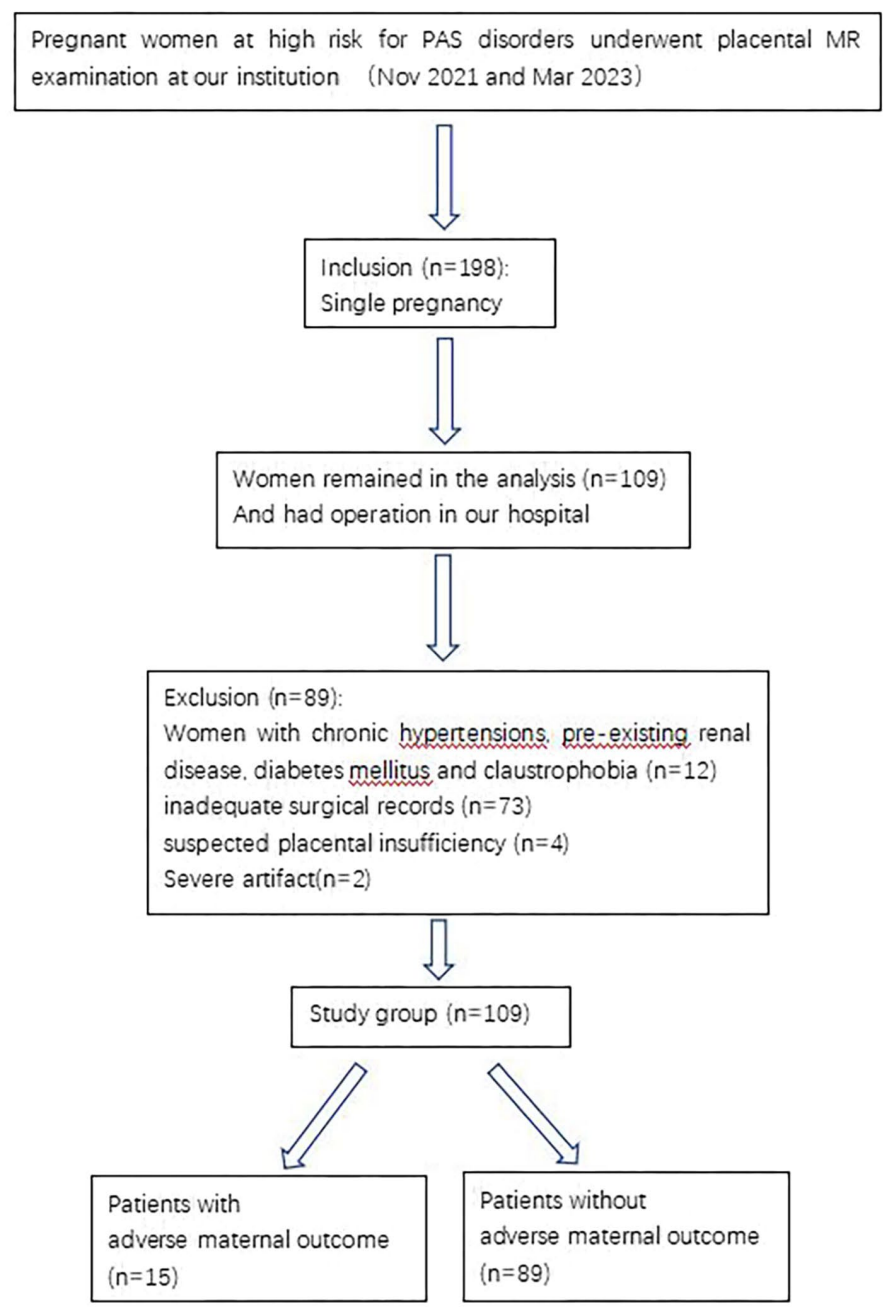
Flowchart of the study design.

The final cohort consisted of 109 patients with a mean maternal age of 31.33 ± 4.56 years (range: 22–45 years) and a mean gestational age of 31 weeks (range: 16–38 weeks) at MRI examination (Figure 1).

### Clinical characteristic analysis

We systematically extracted potential clinical risk factors for PAS disorders from patient medical records, including maternal age, gravidity, parity, number of previous CDs, and number of previous miscarriages. The gestational age at examination, gestational age at delivery, procedure duration, intraoperative blood loss, requirement for blood transfusion, need for hysterectomy, bladder injury requiring repair, and postoperative intensive care unit (ICU) admission were also recorded.

Intraoperative blood loss was measured with reference to the weight of the surgical pads and the volume of the suction jar in the operating room. Massive postpartum hemorrhage was defined as intrapartum or peripartum blood loss > 2,000 mL, prolonged delivery was defined as surgical time > 60 min, minimal bladder repair was defined as the involvement of only the vesicouterine fold or bladder serosa and no or minimal injury to the bladder wall during detachment, and extensive bladder repair was defined as a cystotomy or partial cystectomy.

### MRI protocols

All MRI examinations were conducted on a 1.5 Tesla MR scanner (Aera, Siemens Healthineers, Erlangen, Germany) equipped with a 16-channel body matrix coil. MR sequences, including *Half-Fourier Acquisition Single-shot Turbo Spin-Echo* (HASTE) and *True Fast Imaging with Steady-State Precession* (True-FISP) in the axial, sagittal, and coronal planes, and T1-weighted imaging (T1WI) in the axial and sagittal planes, were used. DWI was performed using a single-shot echo-planar imaging (EPI) sequence with a pair of rectangular diffusion gradient pulses along all three orthogonal axes. The acquisition parameters were as follows: repetition time (TR)/time to echo (TE) of 5,200/83 ms, number of averages of 2, acquisition matrix of 192 × 120, FOV of 390 mm, slice thickness of 5 mm, intersection gap of 5 mm, and parallel imaging acceleration factor of 2. The DWI protocol used 11 *b*-values ranging from 0 to 1,600 s/mm^2^ (b = 0, 50, 100, 150, 200, 400, 600, 800, 1,000, 1,200, and 1,600 s/mm^2^) with a total acquisition time of approximately 7 min and 29 s.

### Image processing

Diffusion-weighted imaging parameters were quantified using IMAge/enGINE (Vusion Tech) (29). Before quantification, routine motion correction was performed on the acquired DWI images. The apparent diffusion coefficient (ADC) was derived using a monoexponential model with *b*-values of 0 and 1,000 s/mm^2^, calculated according to the following equation:


Sb/S0=exp(−b×ADC)

For advanced diffusion characterization, a non-Gaussian model was applied using six *b*-values (0, 400, 800, 1,000, 1,200, and 1,600 s/mm^2^) to calculate the following parameters: MD, representing the corrected apparent diffusion coefficient, and MK, quantifying the degree of diffusion non-Gaussianity. The diffusion kurtosis model was implemented using the following equations (30, 31):


$$\mathrm{Sb}/S0=\exp(-b\times \mathrm{MD}+b^{2}\times MD^{2}\times \frac{\mathrm{MK}}{6})$$

A biexponential model separates the signal decay into a fast diffusion component (perfusion-related) and a slow diffusion component (true molecular diffusion). Eight *b*-values (0, 50, 100, 150, 200, 400, 600, and 800 s/mm^2^) were used to calculate f (perfusion fraction), D (diffusion coefficient), and D* (pseudo-diffusion coefficient) using the following equation (32, 33):


$$\mathrm{Sb}/S0=(1-f)\;\exp(-b\times D)+f\;\exp[-b\times (D+D^{*})]$$

where Sb = signal at a given *b*-value and S0 = signal at b = 0.

All measurements were independently performed by two radiologists with 5 and 8 years of specialized experience in obstetric imaging, respectively. For each case, the regions of interest (ROIs) were manually delineated for every consecutive DWI with b = 0 s/mm^2^ to encompass the entire placental tissue. To minimize the partial volume effect, ROIs were carefully drawn approximately 1–2 mm inside the visible placental margins. These defined ROIs were then automatically propagated to all corresponding diffusion parameter maps for quantitative analysis. ADC, MD, MK, D, D*, and *f* values were systematically calculated. For statistical analysis, the measured values from both readers were averaged to generate the final dataset and were also used to assess the inter-reader agreement. Additionally, intra-reader reproducibility was evaluated using measurements obtained by the first radiologist after a minimum washout period of 1 month to reduce recall bias.

### Statistical analysis

The normality of all data distribution was assessed using the Shapiro–Wilk test. Continuous variables were expressed as mean ± standard deviation (SD) if normally distributed or median (range) if non-normally distributed, while categorical variables were presented as proportions (%). Comparisons between patients with and without adverse maternal outcomes were performed using the Mann–Whitney U-test for continuous variables (including DWI parameters of the whole placenta) and the chi-squared (χ^2^) test for categorical variables. To identify independent risk factors for adverse maternal outcomes in high-risk PAS disorder patients, we conducted univariate and multivariate logistic regression analyses. Significant predictors were further evaluated using receiver operating characteristic (ROC) curve analysis to assess their diagnostic performance. The Z test was used to compare the area under the curves (AUC) for independent ROC curves.

Interparameter correlations (ADC, D, MD, and MK) were assessed using Spearman’s rank correlation analysis. Inter-reader and intra-reader reproducibility for all diffusion parameters were evaluated using the intraclass correlation coefficients (ICCs) with 95% confidence intervals (CIs). A two-tailed *p*-value of < 0.05 was considered statistically significant. All analyses were performed using SPSS Statistics (Version 21.0; IBM Corp., Armonk, NY, United States).

## Results

The distribution of risk factors for PAS disorders among participants was as follows: placenta previa (*n* = 81), previous CDs (*n* = 7), previous miscarriage (*n* = 83), age ≥ 35 years (*n* = 27), surgery for uterine myoma (*n* = 2), *in vitro* fertilization (IVF) (*n* = 1), multiparity (*n* = 11), and uterine anomalies (*n* = 1).

All deliveries were performed as per scheduled procedures. Maternal characteristics are comprehensively presented in Table 1. Among the 109 study participants, adverse outcomes included the following: massive postpartum hemorrhage (*n* = 15, 13.8%), peripartum hysterectomy (*n* = 3, 2.8%), minimal bladder repair (*n* = 9, 8.3%), and prolonged delivery and admission to the ICU (*n* = 7, 6.4%). Patients experiencing adverse maternal outcomes demonstrated significantly higher rates of both placenta previa (*p* = 0.014) and multiple prior cesarean deliveries (*p* = 0.022). A multivariate logistic regression analysis identified the number of prior CDs as an independent clinical predictor of adverse maternal outcomes in high-risk patients with PAS disorders (*p* = 0.018, OR: 3.219).

**Table 1:** Maternal characteristics in the study groups.

Characteristics	Patients without adverse maternal outcomes	Patients with adverse maternal outcomes	*p-*value
Number	94	15	
Age (years)	31.07 ± 4.47	33.0 ± 4.97	0.882
Less than 35	73(77.66%)	9(60%)	0.008
35 or older	21(22.34%)	6(40%)	
Gestational age at examination (weeks)	31(5)	30(3)	0.539
Gestational age at the time of delivery (weeks)	37(3)	36(1)	0.067
Previous cesarean section			0.051
No	37(39.36)	2(13.33)	
Yes	57(60.64)	13(86.67)	
Number of previous cesarean Section			0.022
0	39(41.49%)	2(13.33%)	
1	48(51.06%)	9(60%)	
2 or more	7(7.45%)	4(26.67%)	
Previous miscarriages			0.093
No	25(26.60%)	1(6.67%)	
Yes	69(73.40%)	14(93.33%)	
Number of Previous miscarriages			0.534
0	23(24.47%)	1(6.67%)	
1	26(27.664%)	5(33.33%)	
2 or more	45(47.87%)	9(60%)	
Placenta previa			0.014
No	28(29.79%)	0(0%)	
Yes	66(70.21%)	15(100%)	
Placenta position			0.074
Posterior	40(42.55%)	9(60%)	
Anterior	34(36.17%)	1(6.67%)	
Anterior+posterior	20(21.28%)	5(33.33%)	

Both intra-observer and inter-observer agreement for placental measurements demonstrated excellent reliability, with intra-reader intraclass correlation coefficients (ICCs) ranging from 0.652 to 0.948 and inter-reader ICCs ranging from 0.652 to 0.911 (Table 2). The volumetric assessment of the entire placenta showed particularly strong agreement, with ICCs consistently in the excellent range (>0.60).

**Table 2:** Inter-reader and intra-reader reproducibility for DWI parameters.

Parameters	ICC (95% CI)	
	Inter-reader	Intra-reader
Standard DWI parameters		
ADC mean (×10^−3^mm^2^/s)	0.822 (0.713–0.892)	0.948 (0.902–0.972)
DKI parameters		
MD mean (×10^−3^mm^2^/s)	0.737 (0.589–0.838)	0.818 (0.678–0.901)
MK mean	0.911 (0.853–0.947)	0.947 (0.901–0.972)
IVIM parameters		
f mean (%)	0.715 (0.557–0.823)	0.804 (0.654–0.893)
D mean (×10^−3^mm^2^/s)	0.872 (0.770–0.927)	0.937 (0.881–0.967)
D* mean (×10^−3^mm^2^/s)	0.666 (0.470–0.795)	0.652 (0.426–0.802)

**Figure 2:**
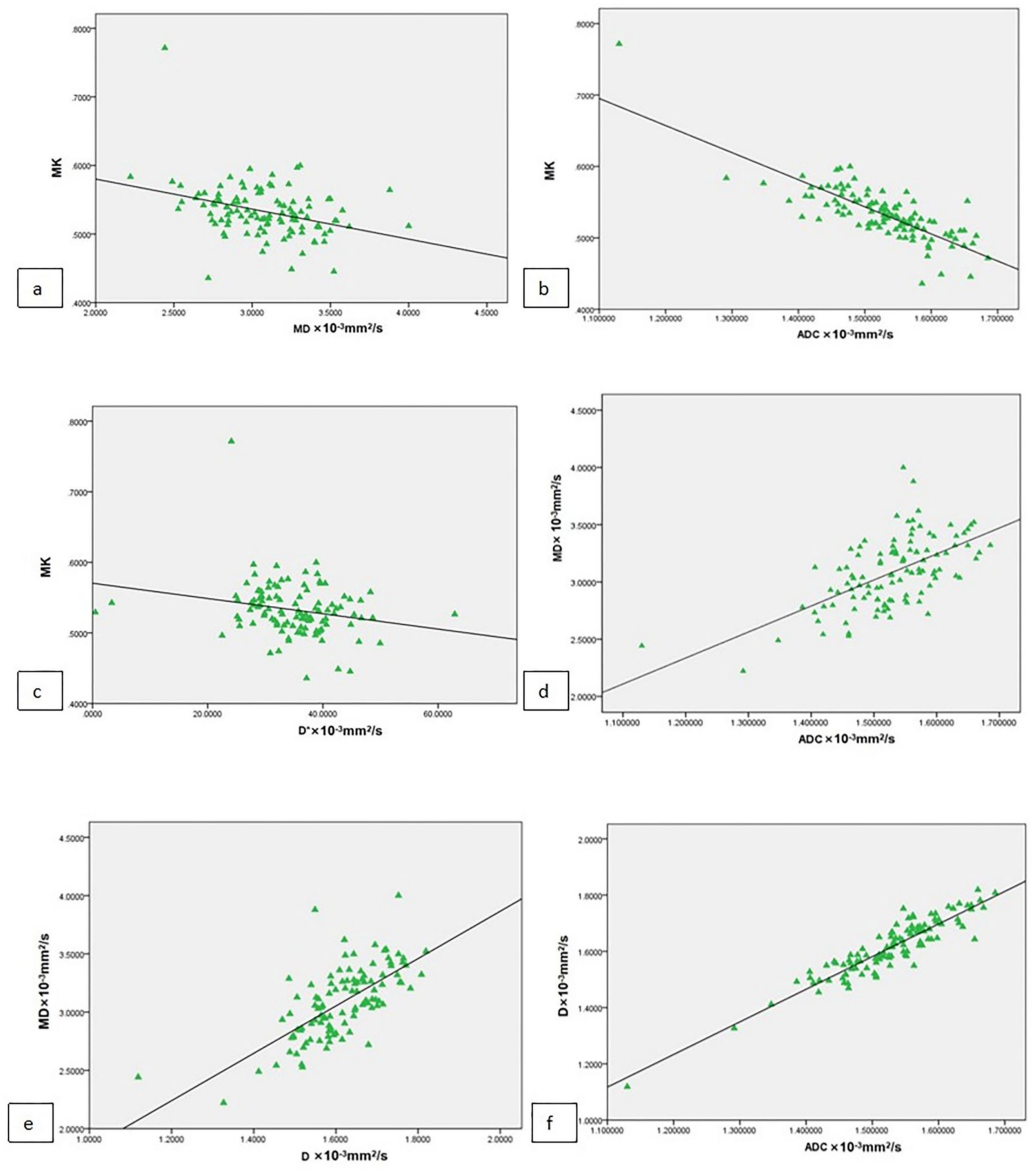
Scatter plots show the correlations among DWI parameters. The plots showed that MK had a negative correlation with ADC, D, and MD **(a–c)**. MD was positively correlated with ADC, and D **(d,e)**, and ADC was positively correlated with D **(f)**.

Diffusion parameter analysis revealed significant correlations among the following measured indices: MK demonstrated negative correlations with ADC, D, and MD (all *p* < 0.0001). MD showed positive correlations with ADC and D (all *p* < 0.0001), and a significant positive correlation was observed between ADC and D (*p* < 0.0001) (Figure 2 and Table 3).

**Table 3:** Correlations among the diffusion-derived parameters.

Parameters	r (95% CI)	*p*-value
MD vs. MK	−0.347	<0.0001
ADC vs. MK	−0.778	<0.0001
D vs. MK	−0.820	<0.0001
ADC vs. MD	0.591	<0.0001
D vs. MD	0.682	<0.0001
D vs. ADC	0.892	<0.0001

**Figure 3:**
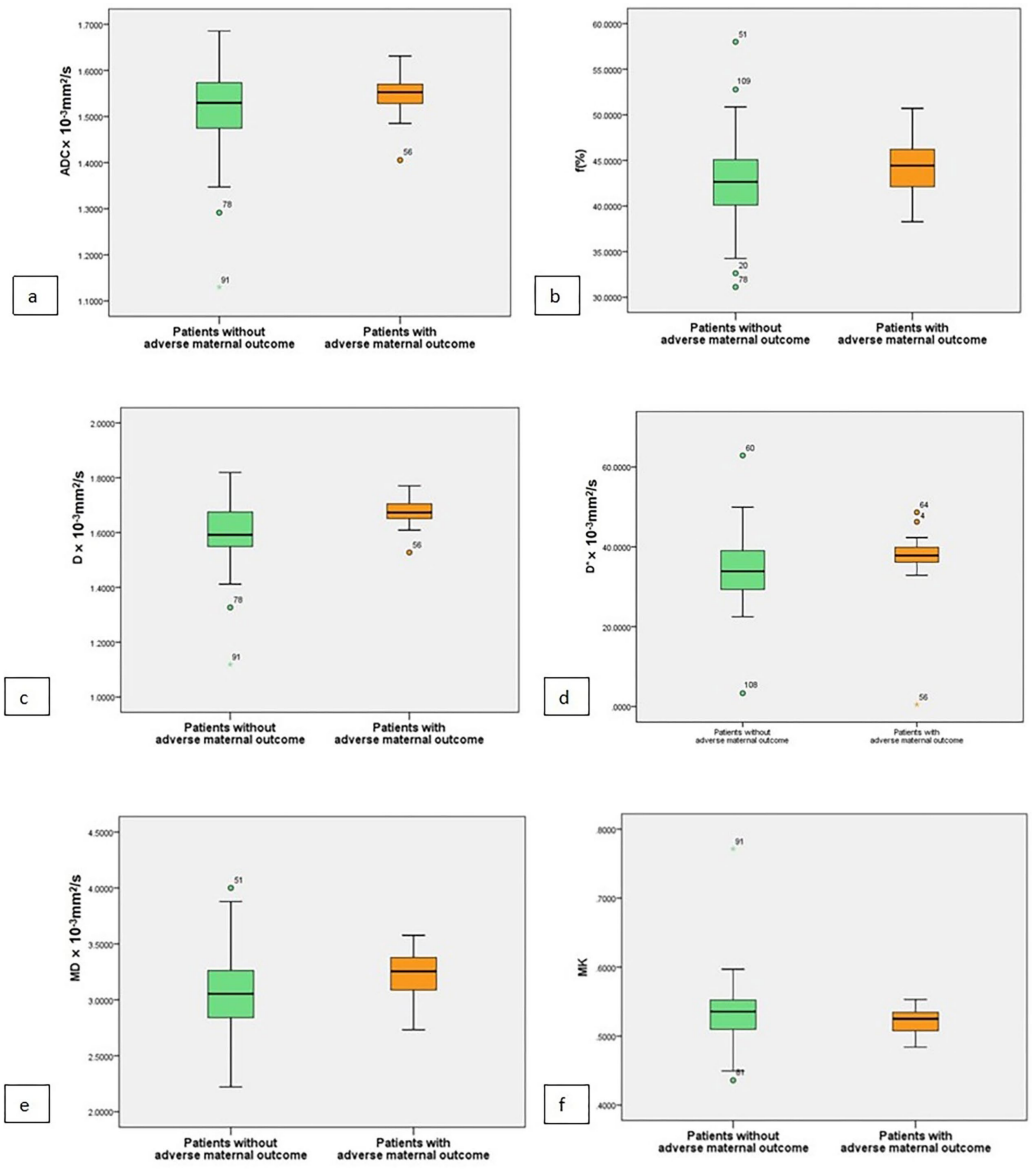
Box and whisker plots of ADC, D, D*, f, MD, and MK for patients with and without adverse maternal outcomes **(a–f)**. The plots showed that D, D*, and MD were significantly higher **(c–e)**, and MK was significantly lower **(f)** in patients with adverse maternal outcomes.

**Figure 4:**
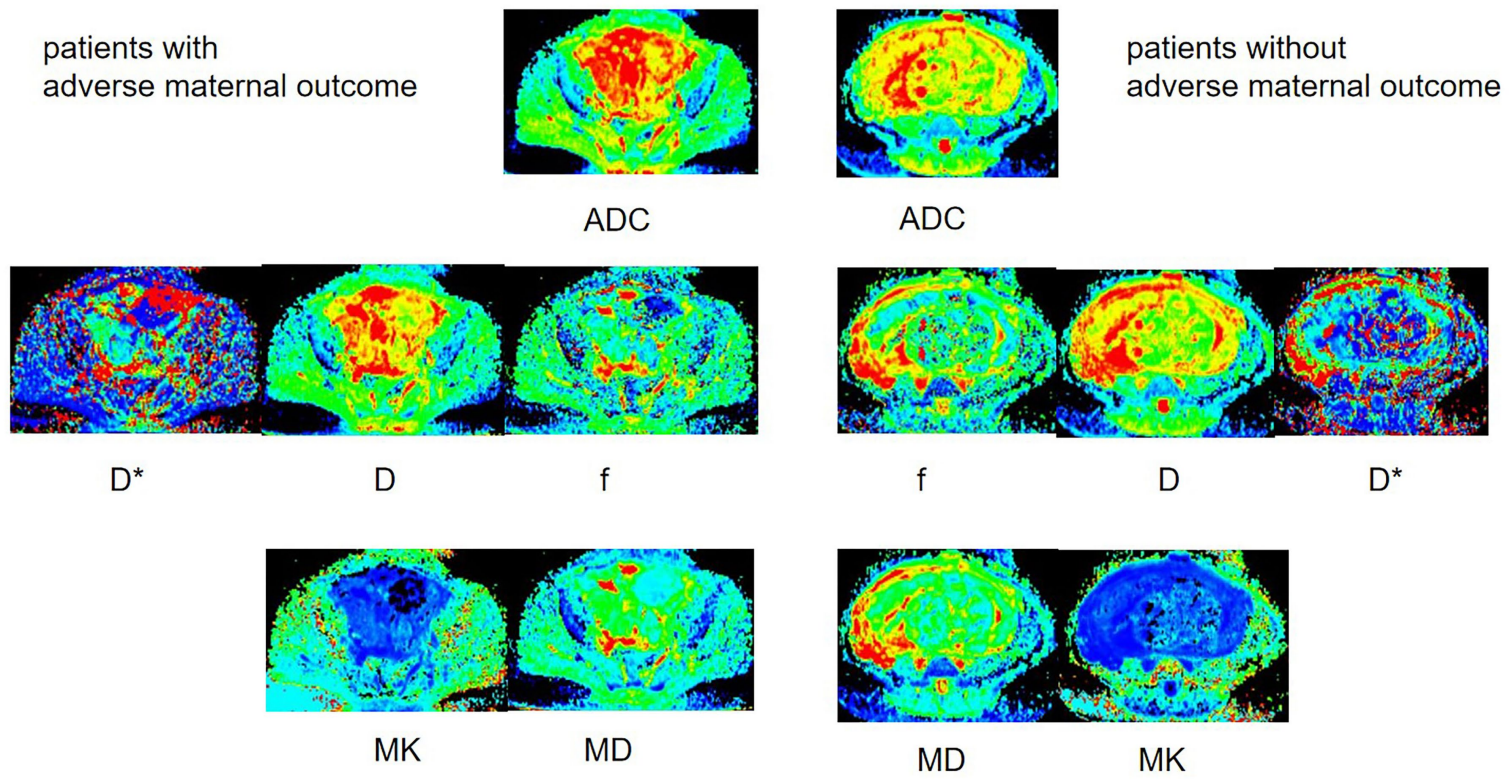
Illustration of DWI parameters in patients with and without adverse maternal outcomes. The figure showed a 33-year-old woman with and a 25-year-old woman without adverse maternal outcomes. Both patients had placenta previa and one prior CD. The figure demonstrated heterogeneous hyperintensity on ADC, D, D*, and MD maps and hypointensity on the MK map.

A comparative analysis of DWI parameters revealed significant differences between outcome groups (Table 4, Figures 3, 4). Patients with adverse maternal outcomes exhibited significantly higher MD (*p* = 0.004), D* (*p* = 0.029), and D values (*p* = 0.024) and significantly lower MK values (*p* = 0.042).

**Table 4:** Comparison of DWI parameters between patients with and without adverse maternal outcomes (*n* = 109).

Parameters	Patients without adverse maternal outcomes	Patients with adverse maternal outcomes	*P*-value
Standard DWI parameters			
ADC mean (×10^−3^mm^2^/s)	1.529(0.90)	1.552(0.05)	0.287
DKI parameters			
MD mean (×10^−3^mm^2^/s)	3.054(0.422)	3.254(0.318)	0.024
MK mean	0.533(0.442)	0.5213(0.029)	0.042
IVIM parameters			
f mean (%)	42.654(5.073%)	44.432(4.916)	0.655
D mean (×10^−3^mm^2^/s)	1.591(0.126)	1.673(0.062)	0.004
D* mean (×10^−3^mm^2^/s)	33.872(9.7642)	37.815(3.986)	0.029

**Figure 5:**
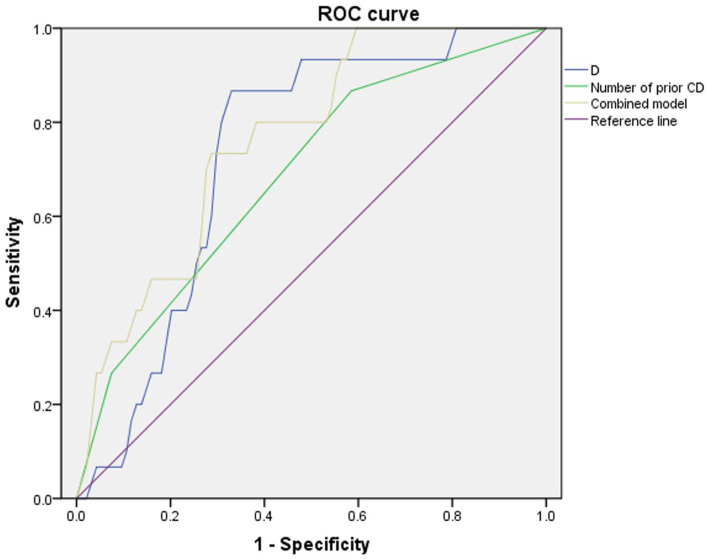
ROC curves for predicting patients with adverse maternal outcomes. A combination of the number of CDs and D showed the best overall performance.

The multivariate logistic regression analysis identified D as an independent predictor of adverse maternal outcomes in high-risk patients for PAS disorders (*p* = 0.014, OR:1.053). Diagnostic performance metrics are detailed in Table 5. Among individual predictors, D demonstrated superior discriminative ability (AUC = 0.730, 95% CI 0.619–0.842) with a cutoff of 1.64×10^−3^ mm2/s, and prior CD frequency showed moderate predictive value (AUC = 0.687; 95% CI 0.545–0.828) with a cutoff of 1. The combined model integrating both D and prior CD history achieved optimal performance, with a sensitivity of 73%, a specificity of 72%, and an AUC of 0.766 (95% CI: 0.654–0.878) (Figure 5). Significant differences were found in the AUCs between the combined model and D (*p* = 0.028) and between the combined model and the number of prior CDs (*p* = 0.013).

**Table 5:** Predictive performance of risk factors for patients with adverse maternal outcome.

Risk factors	AUC	Accuracies (%)	Sensitivities (%)	Specificities (%)	PPV (%)	NPV (%)	*P*-value	95%CI
D	0.730	77.5	87	68	71.68	78.16	0.004	0.619–0.842
Number of CDs	0.687	61.6	86.7	41.5	59.71	75.73	0.021	0.545–0.828
Combination of the number of CDs and D	0.766	72.5	73	72	79.35	75	0.001	0.654–0.878

## Discussion

PAS disorders are well-established risk factors for severe obstetric complications, including massive postpartum hemorrhage, emergent hysterectomy, and urinary tract injury (34). While previous studies have explored conventional MRI signs for predicting clinical outcomes in suspected PAS cases (35–37), the diagnostic reliability of these findings remains limited by interobserver variability and subjective interpretation (38, 39). Moreover, the prognostic value of diffusion-weighted imaging parameters for adverse maternal outcomes has not been conclusively established. To address these limitations, our study used advanced diffusion metrics (DKI and IVIM parameters) for placental evaluation and outcome prediction. Our key findings demonstrate that patients experiencing adverse outcomes (including massive hemorrhage, hysterectomy, bladder injury, prolonged surgery, and ICU admission) exhibited significantly elevated D, D*, and MD values alongside reduced MK values. Among all diffusion parameters, D emerged as an independent predictor of adverse outcomes, and the number of prior cesarean deliveries served as an independent clinical predictor. The combined model integrating both D values and prior CD history showed superior predictive performance for adverse outcomes in high-risk PAS patients.

Massive postpartum hemorrhage (PPH) in PAS disorders typically occurs as part of a complex clinical syndrome rather than an isolated complication. Our observations demonstrate that PPH frequently coexists with bladder wall injury requiring surgical repair, prolonged operative time, peripartum hysterectomy, and ICU admission, reflecting the severe morbidity spectrum associated with advanced PAS. Notably, even minimal bladder involvement in the placenta percreta cases (where placental tissue adheres to the bladder serosa) can precipitate significant bladder wall disruption and life-threatening hemorrhage (40). The clinical ramifications of these complications are substantial. ICU admission significantly prolongs hospitalization and increases healthcare costs. Extended operative duration raises risks associated with prolonged general anesthesia, including maternal respiratory complications. These factors may also contribute to adverse neonatal outcomes (35, 41). Given this cascade of potential complications, accurate antenatal prediction of adverse outcomes becomes paramount for multidisciplinary delivery planning and patient counseling regarding surgical risks.

The IVIM model describes water molecule distribution in a voxel through two distinct compartments: capillary network microcirculation and water molecular diffusion. In this study, parameter D represents pure water diffusion, while D* reflects microcirculatory perfusion effects. In high-risk patients with PAS disorders, blastocyst implantation preferentially occurs at scar tissue sites. This leads to abnormal trophoblastic cell invasion, resulting in either dilation of the myometrial vasculature or pathological neovascularization (42). MRI in placenta percreta demonstrates serosal hypervascularity and an extensive vascular plexus invading the vesicouterine space or parametrial fat, especially with bladder or parametrial involvement (37). Serosal hypervascularity and abnormal intraplacental vascularization may arise from a shared pathological process, likely reflecting aberrant vascular remodeling at the placental-maternal interface (42). The presence of abnormally enlarged arteries, which accommodate significantly higher blood volumes, predisposes to massive hemorrhage upon placental removal. These dilated vessels may also contribute to enhanced extravascular water diffusion within the placental villi and augmented blood flow in fetal capillaries. In cases with adverse maternal outcomes, these hemodynamic alterations likely drive the observed elevations in IVIM parameters (D and D values), reflecting increased water molecule mobility and perfusion.

Diffusional kurtosis imaging (DKI) is a non-Gaussian diffusion model that quantifies the excess kurtosis in the probability distribution of water molecule displacements, providing insights into tissue microstructural complexity beyond conventional diffusion metrics (43). Mean kurtosis (MK) is a dimensionless parameter that quantifies the degree of deviation from Gaussian diffusion behavior, whereas mean diffusivity (MD) represents the corrected apparent diffusion coefficient (ADC). Our study demonstrated an inverse relationship between MK and ADC, D, and MD values, suggesting reduced tissue microstructural complexity with increasing diffusivity. Conversely, MD exhibited strong positive correlations with both ADC and D, reflecting their shared dependence on water molecule mobility. Consistent with prior studies, a significant negative correlation was observed between mean kurtosis (MK) and diffusion parameters (ADC, D, and MD) derived from higher *b*-values (41, 42). Therefore, our study demonstrated significantly elevated mean diffusivity (MD) and reduced mean kurtosis (MK) in patients with adverse maternal outcomes.

Our study identified the IVIM-derived diffusion coefficient (D) as an independent predictor of adverse maternal outcomes among all evaluated DWI parameters. As D reflects pure molecular diffusion independent of microcirculatory effects, its marked elevation suggests substantial increases in extracellular water mobility. Additionally, the number of prior cesarean deliveries (CDs) emerged as an independent clinical predictor of adverse maternal outcomes, consistent with existing evidence demonstrating a strong dose–response relationship between CD history and placenta accreta spectrum (PAS) risk. Specifically, a previous large observational study reported that, among patients with placenta previa, the probability of PAS disorders escalates dramatically from 3% after one CD to 67% after five CDs, highlighting the cumulative impact of uterine scarring on placental pathology (44). Our study demonstrated a direct relationship between the depth of placental invasion and the likelihood of adverse maternal outcomes. To improve clinical risk stratification, we developed a predictive model combining two independent risk factors: (1) number of prior cesarean deliveries (CDs) and (2) IVIM-derived diffusion coefficient (D). This combined approach showed strong diagnostic performance in high-risk PAS patients, with a sensitivity of 73%, a specificity of 72%, a positive predictive value (PPV) of 79.4%, a negative predictive value (NPV) of 75%, and an AUC of 0.766. Our findings demonstrate that the combined use of CD history and diffusion coefficient (D) provides more accurate risk stratification compared to either parameter alone. This powerful combination effectively identifies patients at the highest risk for severe maternal complications, including massive postpartum hemorrhage, repair of the bladder wall, prolonged operation time, hysterectomy, and admission to the ICU. For these high-risk cases, we recommend the implementation of a multidisciplinary team (MDT) approach, incorporating specialists in obstetrical care, urology, interventional radiology, anesthesia, and blood banking. This proactive MDT strategy enables comprehensive preoperative planning and immediate access to critical interventions, significantly reducing maternal morbidity in complex PAS cases.

Several important limitations should be considered when interpreting our findings. First, the retrospective design and small sample size (particularly only 15 cases with adverse outcomes) may introduce selection bias and limit the generalizability of the cutoff values and regression model stability. The modest sample size of complication cases reduces statistical power for detecting subtle but clinically meaningful associations. Further studies with larger sample sizes, including more patients with adverse maternal outcomes, are needed. Second, the DKI protocol in our study was acquired under free breathing, which may decrease the signal-to-noise ratio on parameter maps. Since breath-holding is impractical for pregnant women, we employed a free-breathing acquisition approach. The good inter-observer agreement in our study confirms the feasibility of using this DKI model for placental imaging. Third, while the combined model of CD history and D values demonstrated moderate predictive value (AUC = 0.766), its performance appeared somewhat limited compared with certain MRI morphological signs reported in the literature. However, this comparison requires careful interpretation, as MRI sign evaluation depends heavily on radiologists’ experience, with superior performance typically observed among senior specialists. In contrast, our whole-placenta ROI approach eliminates operator-dependent variability, ensuring more objective and reproducible measurements. These methodological differences suggest that our quantitative model may offer more consistent results across institutions and experience levels, despite slightly lower absolute performance metrics.

In conclusion, our quantitative MRI analysis revealed distinct diffusion patterns associated with adverse maternal outcomes, characterized by significantly elevated D, D*, and MD values, coupled with reduced MK values. These parameters reflect altered placental microstructure and perfusion characteristics in high-risk PAS cases. Importantly, we demonstrated that combining clinical history (number of CDs) with the IVIM-derived diffusion coefficient (D) provides an objective, operator-independent method for predicting adverse outcomes in PAS patients. This integrated approach offers clinicians a valuable tool for risk stratification and preoperative planning in complex placental disorders.

## Data Availability

The raw data supporting the conclusions of this article will be made available by the authors, without undue reservation.
